# Hygeia as Muse

**DOI:** 10.3201/eid1403.032008

**Published:** 2008-03

**Authors:** Polynexi Potter

**Affiliations:** * Centers for Disease Control and Prevention, Atlanta, Georgia, USA

**Keywords:** Henri de Toulouse-Lautrec, lithography, art and science, emerging infectious diseases, At the Moulin Rouge: The Dance, lithographic posters, pycnodysostosis, the Belle Époch, about the cover

**Figure:**
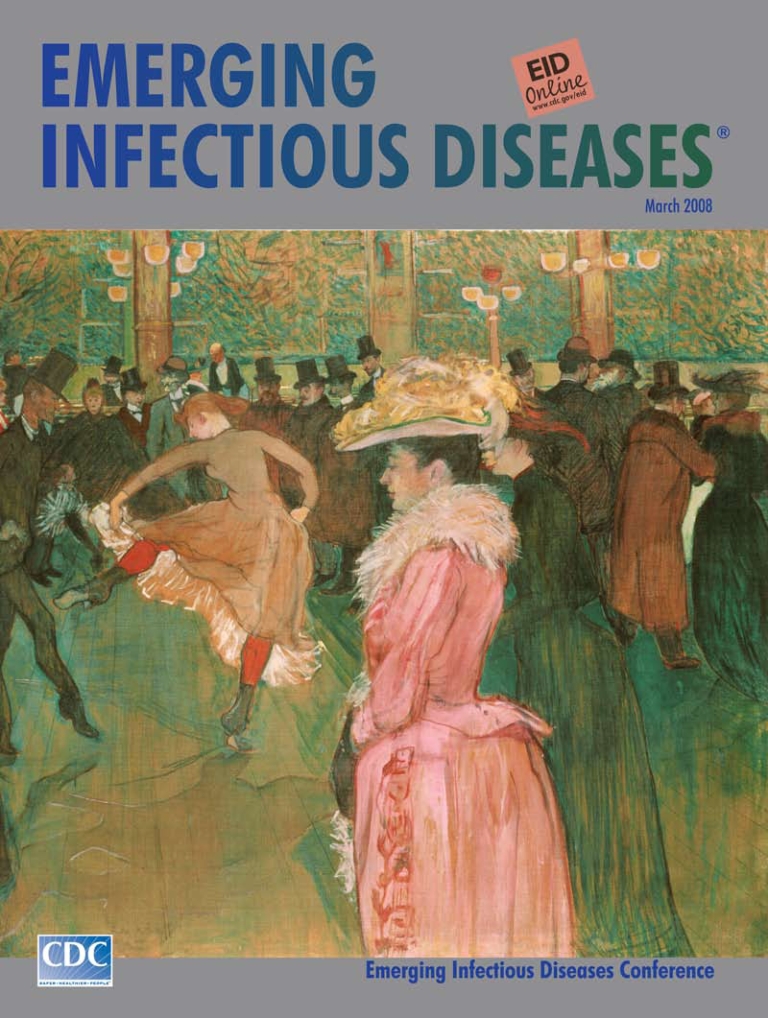
**Henri de Toulouse-Lautrec (1864–1901). At the Moulin Rouge: The Dance (1890).** Oil on canvas (115.6 cm × 149.9 cm). Philadelphia Museum of Art: The Henry P. Mcllhenny Collection in memory of Frances P. Mcllhenny, 1986

“A line will take us hours maybe; / Yet if it does not seem a moment’s thought, / Our stitching and unstitching has been naught,” wrote William Butler Yeats (1865–1939) about the creative process (*1*). For sheer spontaneity, evocativeness, and impeccable draftsmanship, he might have been describing the art of Henri de Toulouse-Lautrec. The artist’s speed at work astonished his friends. Having resolved technical problems in his mind, during the gestation of the image or in countless sketches, photographs, and studies from life, he sang and joked during the brief execution of the work.

Lautrec’s remarkable legacy seems to have started at birth in Albi, one of the oldest cities in France, into a wealthy family with ties to the Counts of Toulouse (*2*). He was an engaging, rambunctious child with precocious wit. At age 4, he wanted to sign the register at his brother’s christening. Reminded that he could not write, he said, “It doesn’t matter, I’ll draw an ox.” That was his earliest known work (*3*). By age 10, he was an inveterate sketcher of people and animals, illustrating everything he touched.

In what would appear an idyllic childhood, signs of trouble, perhaps the underpinnings of aristocratic lineage, started with the death of his young sibling before age 1. Henri, whose parents were first cousins, was also frail. His mother took him out of school and moved to the country, where she devoted herself to his care.

When he was 13, his life began to change: “I fell off a low chair onto the floor and broke my left thigh,” he wrote a friend. After a long convalescence, he could only walk lopsidedly “like a duck.” Fifteen months later, he fell again: “The second fracture was caused by a fall scarcely heavier than the first” (*3*). Henri, it appeared, had some unknown bone disease, a congenital condition, possibly pycnodysostosis (*4*). Despite the best available care and while the rest of his body continued to grow, the legs atrophied. He supported himself on a cane, which was surprisingly short since his trunk and arms were of normal length. Walking caused him pain and embarrassment.

Though limited by physical disability, he remained upbeat. “I am small but I am not a dwarf,” he wrote, “... no urchins have ever bothered me” in the street (*3*). He always wore a hat, even when he painted, “for the light,” he said (*3*), although like his signature beard, it may have concealed bone malformations.

He moved to Paris to study with Léon Bonnat, leading portraitist and later professor at the École des Beaux Arts. This apprenticeship turned him away from academic art: “I want to paint like the primitives, whose painting is as simple as that on a carriage door” (*3*). Later, under Fernand Cormon, he met and befriended Vincent van Gogh, Émile Bernard, and other artists, who sought him out for his openness and originality. Aristide Bruant, legendary balladeer and owner of cabaret Le Mirliton, initiated him to Montmartre: “I am against my will leading a truly Bohemian life and am finding it difficult to accustom myself to this milieu” (*3*).

Montmartre, an area on a hill away from the city, developed a unique personality, energetic and provocative, “outside the law” (*3*). Its dance halls, cabarets, cafés, and circuses held unending fascination. He painted them by day and lived in them by night. “From ten o’clock in the evening until half past twelve,” reported the newspapers, “the Moulin Rouge [red windmill] presents a very Parisian spectacle which husbands may confidently attend accompanied by their wives” (*3*).

Lautrec lived in Montmartre, except for brief visits to Spain where he studied the work of El Greco and Diego Velásquez; Belgium; and England where he met Oscar Wilde and James McNeill Whistler. He exhibited often and was enormously productive, creating before he died at age 36 more than 1,000 paintings and 5,000 drawings. Although he maintained artistic independence, he was well connected. He consulted with Pierre Bonnard, was captivated by the impressionists, collected Japanese prints, and thought an Edgar Degas painting owned by his cousins so compelling, he declared he was always ready to “say his prayers before it” (*3*).

The 1880s and ’90s, the Belle Époch in Europe and around the world, saw unprecedented scientific and technological advancements. Literature and the arts, biology, physics, and psychology were transformed. Theater and music adopted new methods, shocking audiences with their frankness. In Paris, the Moulin de la Galette and the Moulin Rouge offered carnival-like entertainment. Celebrated can-can dancer Jane Avril and Louise Weber, dubbed La Goulue (the glutton, for guzzling drinks), attracted huge crowds with decadent performances. Lautrec with his cherry-wood cane, black-and-white checked trousers, and flat-brimmed bowler hat was a regular fixture.

Lautrec was fascinated by human behavior but painted those who interested him: working women; cabaret proprietors; entertainers, whose brilliant if transient careers he observed dispassionately. His works were restrained, as he thought all art should be, but filled with movement. His sparse palette and bold, assured brushstrokes captured the essence of nightlife, the glare of the stage, the shadows of gaiety, the despair and loneliness of crowds, the plight of the working poor, the physical pain of dancers as well as their agility. On one album of lithographs he wrote, “I saw this,” a phrase borrowed from Francisco Goya’s Disasters of War. The phrase could describe Lautrec’s total artistic output, the life of his era, regulated brothels and all.

Color lithography was new and very popular, and Lautrec adopted it as preferred medium. The technique, practiced by many greats (Alphonse Mucha, Pablo Picasso, Jasper Johns) originated in 1796. Based on the principle that oil and water do not mix, it uses both to form a print on a smooth surface. An image drawn with grease chalk onto a stone is moistened; ink is applied, which sticks to the drawing but not to the stone; inked areas are transferred to paper. Each color requires a different stone and separate pass through the press. Lautrec’s lithographs, though known as posters, were not today’s mass-produced photographic reproductions. They were multiples of small editions, each print individually made, one of a kind, original. “Poster” referred only to large size (*5*).

Publicity posters made by the lithographic technique were a major innovation of Montmartre artists. Lautrec’s first effort and his best, Moulin Rouge: La Goulue, was an overnight sensation, pushing him and La Goulue to stardom. The critics took notice. Notoriety encouraged him to continue in the medium, introducing such innovations as spattered paint to simulate the aura of nightclubs and radical placement of figures and objects to achieve unprecedented immediacy. He created more than 300 lithographs, among the finest ever produced.

At the Moulin Rouge: The Dance (on this month’s cover) epitomizes bourgeois gaiety in a luxurious establishment. This large multiple figure composition exemplifies Lautrec’s vivid contrasting colors and meticulous execution. Dancing on the left is Jacques Renaudin, nicknamed Valentin le désossé (boneless Valentin) because of his rubbery limbs. Gathered in back, left of the waiter, a group of friends, among them Jane Avril, a photographer, some painters; in the center, a professional dancer; mingling with the crowd, colorful women ready to offer their hearts at the right price. The viewer is virtually in the painting.

Amusement, fanatically pursued by the crowds in dance halls and by Lautrec himself, at times, equals living on the edge. For him, excess was compounded by disability and emotional isolation, as well as by the plagues of his time, syphilis (*6*) and possibly tuberculosis: “Visibly, before the very eyes of his friends, he began to burn himself out, slowly at first, then with ever increasing speed” (*3*). He continued to grow as an artist, his style evolving up to the time of death.

The need to gather in large venues to see and be seen and to exchange ideas is not limited to Paris cafés and private clubs. This need, to break barriers, showcase new work, and focus on the problems of humanity, lives on today in scientific conferences. There, ideas mingle with personalities, and bit by bit, solutions are worked out for as the poet put it, “It’s certain there is no fine thing / Since Adam’s fall but needs much labouring” (*1*).

Hygeia is a capricious muse. She eludes the compromised artist toiling in pain and without physical charm but inspires the globe-trotting scientist gathering in today’s venues to blast conventional wisdom and seek solutions to emerging infectious disease.
